# *N*-Benzyl-4-methyl-*N*-(4-methyl­phen­yl)benzene­sulfonamide

**DOI:** 10.1107/S1600536811028522

**Published:** 2011-07-23

**Authors:** Komal Faryal, Muhammad Akhyar Farrukh, Fahim Ashraf Qureshi, Saba Ahmad, Ahmad Adnan, Mehmet Akkurt

**Affiliations:** a Department of Chemistry, Government College University, Lahore 54000, Pakistan; b Department of Physics, Faculty of Sciences, Erciyes University, 38039 Kayseri, Turkey

## Abstract

In the title mol­ecule, C_21_H_21_NO_2_S, the phenyl ring makes the dihedral angles of 74.13 (11) and 80.16 (11)° with the two benzene rings, which are inclined at an angle of 43.73 (10)° with respect to each other. In the crystal, mol­ecules are linked by inter­molecular C—H⋯O hydrogen bonds along the [010] direction. In addition, a weak C—H⋯π (arene) inter­action is observed.

## Related literature

For background and the biological and chemical importance of sulfonamides, see: Hung & Hwang (2007 ▶); Burkhart & Burkhart (2009 ▶); Griffiths-Jones *et al.* (2006 ▶). For related structures, see: Ahmad *et al.* (2011*a* ▶,*b* ▶).
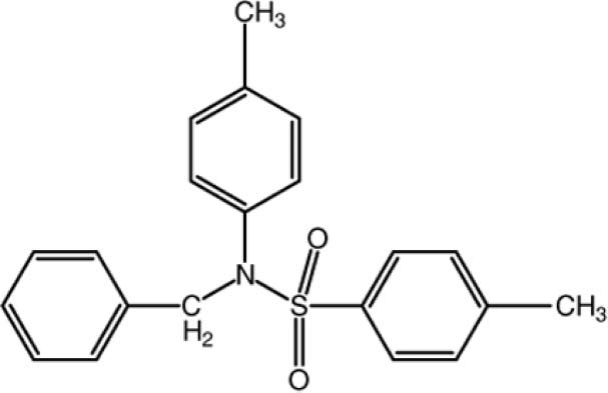


## Experimental

### 

#### Crystal data

C_21_H_21_NO_2_S
*M* *_r_* = 351.46
Monoclinic, 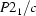

*a* = 9.7089 (6) Å
*b* = 11.5973 (5) Å
*c* = 16.7661 (9) Å
β = 97.691 (2)°
*V* = 1870.83 (17) Å^3^
*Z* = 4
Mo *K*α radiation
μ = 0.19 mm^−1^
*T* = 296 K
0.85 × 0.13 × 0.13 mm

#### Data collection

Bruker APEXII CCD diffractometer
11456 measured reflections
4574 independent reflections
2964 reflections with *I* > 2σ(*I*)
*R*_int_ = 0.023

#### Refinement

*R*[*F* ^2^ > 2σ(*F* ^2^)] = 0.047
*wR*(*F* ^2^) = 0.133
*S* = 1.03
4574 reflections
228 parameters
H-atom parameters constrained
Δρ_max_ = 0.20 e Å^−3^
Δρ_min_ = −0.24 e Å^−3^

### 

Data collection: *APEX2* (Bruker, 2007 ▶); cell refinement: *SAINT* (Bruker, 2007 ▶); data reduction: *SAINT*; program(s) used to solve structure: *SHELXS97* (Sheldrick, 2008 ▶); program(s) used to refine structure: *SHELXL97* (Sheldrick, 2008 ▶); molecular graphics: *ORTEP-3 for Windows* (Farrugia, 1997 ▶); software used to prepare material for publication: *WinGX* (Farrugia, 1999 ▶) and *PLATON* (Spek, 2009 ▶).

## Supplementary Material

Crystal structure: contains datablock(s) global, I. DOI: 10.1107/S1600536811028522/si2365sup1.cif

Structure factors: contains datablock(s) I. DOI: 10.1107/S1600536811028522/si2365Isup2.hkl

Supplementary material file. DOI: 10.1107/S1600536811028522/si2365Isup3.cml

Additional supplementary materials:  crystallographic information; 3D view; checkCIF report

## Figures and Tables

**Table 1:** Hydrogen-bond geometry (Å, °) *Cg*2 is the centroid of the C8–C13 benzene ring.

*D*—H⋯*A*	*D*—H	H⋯*A*	*D*⋯*A*	*D*—H⋯*A*
C21—H21⋯O2^i^	0.93	2.59	3.496 (2)	166
C14—H14*B*⋯*Cg*2^ii^	0.96	2.98	3.540 (2)	118

Symmetry codes: (i) 
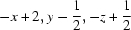
; (ii) 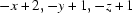.

**Fig. 1.**
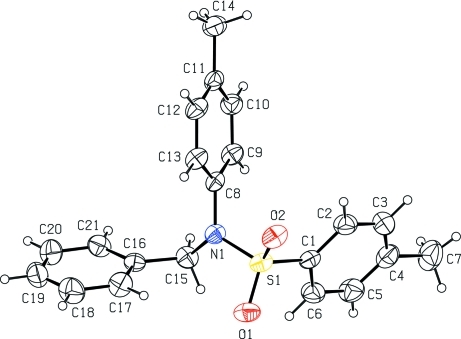
The molecule of the title compound with the atom numbering scheme. Displacement ellipsoids for non-H atoms are drawn at the 30% probability level.

**Fig. 2.**
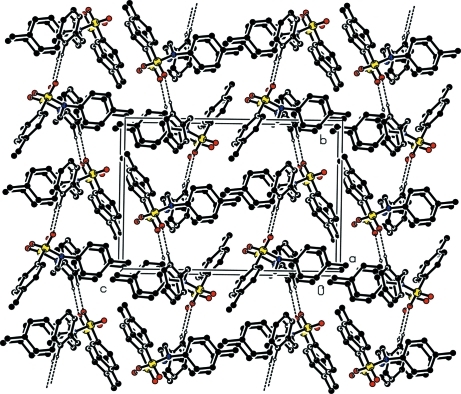
Crystal packing of (I) and the intermolecular C—H···O interactions between the molecules viewed along the a axis. H atoms not involved in hydrogen bonds (dashed lines) have been omitted for clarity.

## supplementary crystallographic information

### Comment

Sulfonamides are compounds of both biological and chemical importance which are widely used in veterinary medicine (Hung & Hwang, 2007). They were developed in the 1930's and were the first effective antimicrobial agent for systemic administration (Burkhart & Burkhart, 2009). Sulfonamides represent an important chemotype for medicinal chemistry. They are not only important in drugs with representation of the largest class of antimicrobial agents but also they form the starting point for many classes of drugs including diuretics, antidiabetic drugs, and antihypertensives (Griffiths-Jones *et al.*, 2006). They have bacteriostatic properties and are effective systematic drug used for humans (Ahmad *et al.*, 2011*a*).

As a contribution to a structural study of sulfonamide derivatives (Ahmad *et al.*, 2011*a*, Ahmad *et al.*, 2011*b*), we report here the title compound, *N*-benzyl-4-methyl-*N*-(4-methylphenyl)benzenesulfonamide, (I).

As shown in Fig. 1, the S1 atom in (I) has a distorted tetrahedral geometry. The largest deviation is in the angle O1—S1—O2 [120.09 (9)°]. The phenyl ring (C16–C21) forms the dihedral angles of 74.13 (11) and 80.16 (11)° with the two benzene rings (C1–C6 and C8–C13). The dihedral angle between the two benzene rings is 43.73 (10)°.

In the crystal structure of (I), neighbouring molecules are connected by intermolecular C–H···O hydrogen bonds (Table 1, Fig. 2) along the *b* axis. The structure is further stabilized by C–H···π (arene) interactions (Table 1).

### Experimental

5 m*M* of *p*-toluidine was dissolved in 20 ml of distilled water then 5 m*M* of benzyl chloride was added. The reaction mixture was stirred properly and 5 m*M* of *p*-toluenesulfonyl chloride was added. The mixture was stirred for about 1–2 h and the pH was maintained 8–10 using 3% solution of Na_2_CO_3_. The reaction was monitored by TLC. The product obtained was filtered and the precipitate was washed with distilled water, dried and recrystallized using methanol.

### Refinement

H atoms were positioned geometrically with C—H = 0.93–0.97 Å and allowed to ride on their parent atoms, with *U*_iso_(H) = 1.2 or 1.5*U*_eq_(C).

### Crystal data

**Table:**

C_21_H_21_NO_2_S	*F*(000) = 744
*M**_r_* = 351.46	*D*_x_ = 1.248 Mg m^−^^3^
Monoclinic, *P*2_1_/*c*	Mo *K*α radiation, λ = 0.71073 Å
Hall symbol: -P 2ybc	Cell parameters from 3286 reflections
*a* = 9.7089 (6) Å	θ = 2.5–24.9°
*b* = 11.5973 (5) Å	µ = 0.19 mm^−^^1^
*c* = 16.7661 (9) Å	*T* = 296 K
β = 97.691 (2)°	Prism, colourless
*V* = 1870.83 (17) Å^3^	0.85 × 0.13 × 0.13 mm
*Z* = 4	

### Data collection

**Table:**

Bruker APEXII CCD diffractometer	2964 reflections with *I* > 2σ(*I*)
Radiation source: sealed tube	*R*_int_ = 0.023
graphite	θ_max_ = 28.3°, θ_min_ = 2.9°
φ and ω scans	*h* = −11→12
11456 measured reflections	*k* = −14→9
4574 independent reflections	*l* = −22→22

### Refinement

**Table:**

Refinement on *F*^2^	Primary atom site location: structure-invariant direct methods
Least-squares matrix: full	Secondary atom site location: difference Fourier map
*R*[*F*^2^ > 2σ(*F*^2^)] = 0.047	Hydrogen site location: inferred from neighbouring sites
*wR*(*F*^2^) = 0.133	H-atom parameters constrained
*S* = 1.03	*w* = 1/[σ^2^(*F*_o_^2^) + (0.0588*P*)^2^ + 0.2963*P*] where *P* = (*F*_o_^2^ + 2*F*_c_^2^)/3
4574 reflections	(Δ/σ)_max_ < 0.001
228 parameters	Δρ_max_ = 0.20 e Å^−^^3^
0 restraints	Δρ_min_ = −0.24 e Å^−^^3^

### Special details

**Table:**

Geometry. Bond distances, angles *etc*. have been calculated using the rounded fractional coordinates. All su's are estimated from the variances of the (full) variance-covariance matrix. The cell e.s.d.'s are taken into account in the estimation of distances, angles and torsion angles
Refinement. Refinement on *F*^2^ for ALL reflections except those flagged by the user for potential systematic errors. Weighted *R*-factors *wR* and all goodnesses of fit *S* are based on *F*^2^, conventional *R*-factors *R* are based on *F*, with *F* set to zero for negative *F*^2^. The observed criterion of *F*^2^ > σ(*F*^2^) is used only for calculating -*R*-factor-obs *etc*. and is not relevant to the choice of reflections for refinement. *R*-factors based on *F*^2^ are statistically about twice as large as those based on *F*, and *R*-factors based on ALL data will be even larger.

### Fractional atomic coordinates and isotropic or equivalent isotropic displacement parameters (Å^2^)

**Table:**

	*x*	*y*	*z*	*U*_iso_*/*U*_eq_	
S1	0.89447 (6)	0.62917 (4)	0.14182 (3)	0.0546 (2)	
O1	0.98019 (17)	0.65672 (12)	0.08157 (8)	0.0726 (5)	
O2	0.81350 (16)	0.71695 (11)	0.17304 (8)	0.0683 (5)	
N1	0.99846 (16)	0.57740 (12)	0.21798 (9)	0.0519 (5)	
C1	0.7808 (2)	0.51881 (16)	0.10313 (10)	0.0527 (6)	
C2	0.6492 (2)	0.50937 (19)	0.12566 (13)	0.0687 (8)	
C3	0.5614 (2)	0.4236 (2)	0.09295 (14)	0.0780 (9)	
C4	0.6002 (3)	0.34620 (19)	0.03843 (13)	0.0733 (8)	
C5	0.7323 (3)	0.3563 (2)	0.01758 (14)	0.0784 (9)	
C6	0.8220 (2)	0.44090 (19)	0.04879 (12)	0.0697 (8)	
C7	0.5014 (3)	0.2542 (2)	0.00171 (18)	0.1051 (12)	
C8	0.94537 (19)	0.56548 (14)	0.29392 (10)	0.0477 (5)	
C9	0.8894 (2)	0.46311 (15)	0.31576 (11)	0.0574 (6)	
C10	0.8403 (2)	0.45394 (17)	0.38906 (12)	0.0610 (7)	
C11	0.8447 (2)	0.54630 (18)	0.44177 (10)	0.0559 (6)	
C12	0.9021 (2)	0.64773 (17)	0.41902 (11)	0.0610 (7)	
C13	0.9533 (2)	0.65776 (16)	0.34641 (11)	0.0572 (6)	
C14	0.7875 (2)	0.5353 (2)	0.52065 (12)	0.0747 (8)	
C15	1.1138 (2)	0.50075 (16)	0.20267 (12)	0.0583 (7)	
C16	1.2518 (2)	0.54652 (15)	0.23897 (11)	0.0501 (6)	
C17	1.2928 (3)	0.65709 (18)	0.22271 (14)	0.0722 (8)	
C18	1.4232 (3)	0.6972 (2)	0.25410 (17)	0.0871 (10)	
C19	1.5127 (3)	0.6278 (3)	0.30155 (16)	0.0857 (10)	
C20	1.4722 (3)	0.5193 (3)	0.31869 (16)	0.0839 (10)	
C21	1.3433 (2)	0.47864 (18)	0.28786 (13)	0.0656 (8)	
H2	0.62040	0.56070	0.16270	0.0820*	
H3	0.47300	0.41800	0.10830	0.0940*	
H5	0.76130	0.30410	−0.01880	0.0940*	
H6	0.91030	0.44580	0.03340	0.0840*	
H7A	0.52650	0.18140	0.02680	0.1580*	
H7B	0.50670	0.24900	−0.05490	0.1580*	
H7C	0.40830	0.27380	0.00990	0.1580*	
H9	0.88460	0.40010	0.28120	0.0690*	
H10	0.80330	0.38410	0.40340	0.0730*	
H12	0.90650	0.71090	0.45340	0.0730*	
H13	0.99310	0.72670	0.33280	0.0690*	
H14A	0.82210	0.59730	0.55570	0.1120*	
H14B	0.81600	0.46300	0.54530	0.1120*	
H14C	0.68780	0.53870	0.51120	0.1120*	
H15A	1.11300	0.49220	0.14510	0.0700*	
H15B	1.09980	0.42510	0.22490	0.0700*	
H17	1.23230	0.70510	0.19040	0.0870*	
H18	1.44980	0.77180	0.24270	0.1040*	
H19	1.60070	0.65430	0.32210	0.1030*	
H20	1.53270	0.47210	0.35170	0.1010*	
H21	1.31760	0.40420	0.30020	0.0790*	

### Atomic displacement parameters (Å^2^)

**Table:**

	*U*^11^	*U*^22^	*U*^33^	*U*^12^	*U*^13^	*U*^23^
S1	0.0717 (3)	0.0488 (3)	0.0443 (2)	0.0064 (2)	0.0110 (2)	0.0024 (2)
O1	0.0941 (11)	0.0714 (9)	0.0561 (8)	−0.0079 (8)	0.0235 (8)	0.0082 (7)
O2	0.0904 (11)	0.0539 (8)	0.0591 (8)	0.0215 (7)	0.0047 (8)	0.0001 (6)
N1	0.0557 (9)	0.0520 (8)	0.0482 (8)	0.0090 (7)	0.0078 (7)	−0.0024 (7)
C1	0.0624 (12)	0.0568 (10)	0.0387 (8)	0.0080 (9)	0.0062 (8)	0.0028 (8)
C2	0.0720 (15)	0.0785 (14)	0.0575 (12)	0.0078 (12)	0.0162 (11)	−0.0034 (10)
C3	0.0619 (14)	0.0962 (17)	0.0762 (15)	−0.0046 (13)	0.0099 (12)	0.0096 (14)
C4	0.0850 (17)	0.0733 (14)	0.0577 (12)	−0.0063 (13)	−0.0050 (12)	0.0072 (11)
C5	0.0938 (18)	0.0759 (15)	0.0663 (14)	−0.0070 (13)	0.0140 (13)	−0.0197 (11)
C6	0.0716 (14)	0.0768 (14)	0.0637 (12)	−0.0040 (12)	0.0205 (11)	−0.0152 (11)
C7	0.109 (2)	0.099 (2)	0.099 (2)	−0.0341 (17)	−0.0168 (17)	0.0030 (16)
C8	0.0517 (10)	0.0474 (9)	0.0421 (8)	0.0067 (8)	−0.0009 (8)	−0.0019 (7)
C9	0.0710 (13)	0.0470 (10)	0.0533 (10)	−0.0028 (9)	0.0047 (10)	−0.0074 (8)
C10	0.0676 (13)	0.0581 (11)	0.0573 (11)	−0.0048 (10)	0.0082 (10)	0.0045 (9)
C11	0.0520 (11)	0.0688 (12)	0.0447 (9)	0.0146 (10)	−0.0011 (8)	0.0039 (9)
C12	0.0743 (14)	0.0598 (12)	0.0472 (10)	0.0123 (10)	0.0015 (10)	−0.0097 (8)
C13	0.0732 (13)	0.0454 (10)	0.0516 (10)	0.0031 (9)	0.0032 (10)	−0.0030 (8)
C14	0.0734 (15)	0.0983 (17)	0.0530 (11)	0.0207 (13)	0.0112 (11)	0.0071 (11)
C15	0.0619 (13)	0.0487 (10)	0.0656 (12)	0.0056 (9)	0.0132 (10)	−0.0092 (9)
C16	0.0565 (11)	0.0463 (9)	0.0497 (9)	0.0024 (8)	0.0153 (9)	−0.0028 (8)
C17	0.0826 (17)	0.0550 (12)	0.0773 (14)	−0.0043 (11)	0.0041 (13)	0.0050 (10)
C18	0.093 (2)	0.0713 (15)	0.0988 (19)	−0.0289 (15)	0.0195 (17)	−0.0106 (14)
C19	0.0598 (15)	0.114 (2)	0.0833 (17)	−0.0113 (15)	0.0095 (13)	−0.0232 (16)
C20	0.0652 (16)	0.1031 (19)	0.0815 (16)	0.0104 (14)	0.0031 (13)	0.0043 (14)
C21	0.0638 (14)	0.0626 (12)	0.0725 (13)	0.0068 (11)	0.0168 (11)	0.0092 (10)

### Geometric parameters (Å, °)

**Table:**

S1—O1	1.4283 (16)	C18—C19	1.360 (4)
S1—O2	1.4278 (15)	C19—C20	1.360 (5)
S1—N1	1.6326 (16)	C20—C21	1.372 (4)
S1—C1	1.7565 (19)	C2—H2	0.9300
N1—C8	1.443 (2)	C3—H3	0.9300
N1—C15	1.479 (2)	C5—H5	0.9300
C1—C2	1.384 (3)	C6—H6	0.9300
C1—C6	1.380 (3)	C7—H7A	0.9600
C2—C3	1.375 (3)	C7—H7B	0.9600
C3—C4	1.369 (3)	C7—H7C	0.9600
C4—C5	1.379 (4)	C9—H9	0.9300
C4—C7	1.510 (4)	C10—H10	0.9300
C5—C6	1.369 (3)	C12—H12	0.9300
C8—C9	1.376 (2)	C13—H13	0.9300
C8—C13	1.381 (2)	C14—H14A	0.9600
C9—C10	1.381 (3)	C14—H14B	0.9600
C10—C11	1.386 (3)	C14—H14C	0.9600
C11—C12	1.377 (3)	C15—H15A	0.9700
C11—C14	1.507 (3)	C15—H15B	0.9700
C12—C13	1.380 (3)	C17—H17	0.9300
C15—C16	1.493 (3)	C18—H18	0.9300
C16—C17	1.381 (3)	C19—H19	0.9300
C16—C21	1.373 (3)	C20—H20	0.9300
C17—C18	1.385 (4)	C21—H21	0.9300
			
O1—S1—O2	120.09 (9)	C4—C3—H3	119.00
O1—S1—N1	106.11 (9)	C4—C5—H5	119.00
O1—S1—C1	107.37 (9)	C6—C5—H5	119.00
O2—S1—N1	106.78 (8)	C1—C6—H6	120.00
O2—S1—C1	107.78 (9)	C5—C6—H6	120.00
N1—S1—C1	108.25 (8)	C4—C7—H7A	109.00
S1—N1—C8	117.96 (12)	C4—C7—H7B	109.00
S1—N1—C15	119.26 (12)	C4—C7—H7C	109.00
C8—N1—C15	117.64 (14)	H7A—C7—H7B	110.00
S1—C1—C2	120.94 (15)	H7A—C7—H7C	110.00
S1—C1—C6	119.84 (15)	H7B—C7—H7C	109.00
C2—C1—C6	119.22 (18)	C8—C9—H9	120.00
C1—C2—C3	119.59 (19)	C10—C9—H9	120.00
C2—C3—C4	121.9 (2)	C9—C10—H10	119.00
C3—C4—C5	117.5 (2)	C11—C10—H10	119.00
C3—C4—C7	121.3 (2)	C11—C12—H12	119.00
C5—C4—C7	121.2 (2)	C13—C12—H12	119.00
C4—C5—C6	122.0 (2)	C8—C13—H13	120.00
C1—C6—C5	119.7 (2)	C12—C13—H13	120.00
N1—C8—C9	121.21 (15)	C11—C14—H14A	110.00
N1—C8—C13	119.47 (15)	C11—C14—H14B	109.00
C9—C8—C13	119.31 (16)	C11—C14—H14C	109.00
C8—C9—C10	119.95 (17)	H14A—C14—H14B	109.00
C9—C10—C11	121.56 (18)	H14A—C14—H14C	109.00
C10—C11—C12	117.56 (17)	H14B—C14—H14C	109.00
C10—C11—C14	120.72 (18)	N1—C15—H15A	109.00
C12—C11—C14	121.72 (18)	N1—C15—H15B	109.00
C11—C12—C13	121.55 (18)	C16—C15—H15A	109.00
C8—C13—C12	120.05 (17)	C16—C15—H15B	109.00
N1—C15—C16	112.00 (15)	H15A—C15—H15B	108.00
C15—C16—C17	121.09 (19)	C16—C17—H17	120.00
C15—C16—C21	120.87 (17)	C18—C17—H17	120.00
C17—C16—C21	118.0 (2)	C17—C18—H18	120.00
C16—C17—C18	120.7 (2)	C19—C18—H18	120.00
C17—C18—C19	120.2 (2)	C18—C19—H19	120.00
C18—C19—C20	119.4 (3)	C20—C19—H19	120.00
C19—C20—C21	120.9 (3)	C19—C20—H20	120.00
C16—C21—C20	120.8 (2)	C21—C20—H20	120.00
C1—C2—H2	120.00	C16—C21—H21	120.00
C3—C2—H2	120.00	C20—C21—H21	120.00
C2—C3—H3	119.00		
			
O1—S1—N1—C8	166.82 (12)	C2—C3—C4—C5	0.6 (3)
O2—S1—N1—C8	37.62 (14)	C7—C4—C5—C6	178.4 (2)
C1—S1—N1—C8	−78.19 (14)	C3—C4—C5—C6	−0.9 (3)
O1—S1—N1—C15	−39.11 (15)	C4—C5—C6—C1	0.3 (3)
O2—S1—N1—C15	−168.32 (13)	N1—C8—C13—C12	179.59 (17)
C1—S1—N1—C15	75.88 (15)	N1—C8—C9—C10	179.53 (17)
O1—S1—C1—C2	−149.26 (16)	C13—C8—C9—C10	0.8 (3)
O2—S1—C1—C2	−18.57 (18)	C9—C8—C13—C12	−1.7 (3)
N1—S1—C1—C2	96.58 (17)	C8—C9—C10—C11	0.5 (3)
O1—S1—C1—C6	30.00 (18)	C9—C10—C11—C14	178.56 (18)
O2—S1—C1—C6	160.69 (15)	C9—C10—C11—C12	−1.0 (3)
N1—S1—C1—C6	−84.16 (17)	C10—C11—C12—C13	0.1 (3)
S1—N1—C8—C9	94.38 (18)	C14—C11—C12—C13	−179.43 (18)
C15—N1—C8—C9	−60.1 (2)	C11—C12—C13—C8	1.2 (3)
S1—N1—C15—C16	122.31 (15)	N1—C15—C16—C17	−53.5 (2)
C15—N1—C8—C13	118.60 (19)	N1—C15—C16—C21	128.29 (19)
S1—N1—C8—C13	−86.91 (19)	C15—C16—C17—C18	−177.4 (2)
C8—N1—C15—C16	−83.55 (19)	C21—C16—C17—C18	0.9 (3)
C6—C1—C2—C3	−0.7 (3)	C15—C16—C21—C20	177.5 (2)
S1—C1—C2—C3	178.54 (17)	C17—C16—C21—C20	−0.8 (3)
C2—C1—C6—C5	0.5 (3)	C16—C17—C18—C19	0.0 (4)
S1—C1—C6—C5	−178.78 (17)	C17—C18—C19—C20	−0.9 (4)
C1—C2—C3—C4	0.2 (3)	C18—C19—C20—C21	0.9 (4)
C2—C3—C4—C7	−178.6 (2)	C19—C20—C21—C16	−0.1 (4)

### Hydrogen-bond geometry (Å, °)

**Table:**

Cg2 is the centroid of the C8–C13 benzene ring.

**Table:**

*D*—H···*A*	*D*—H	H···*A*	*D*···*A*	*D*—H···*A*
C2—H2···O2	0.93	2.60	2.938 (3)	102
C15—H15A···O1	0.97	2.46	2.894 (2)	107
C21—H21···O2^i^	0.93	2.59	3.496 (2)	166
C14—H14B···Cg2^ii^	0.96	2.98	3.540 (2)	118

Symmetry codes: (i) −*x*+2, *y*−1/2, −*z*+1/2; (ii) −*x*+2, −*y*+1, −*z*+1.
